# Paracoccidioidomycosis of the testis

**DOI:** 10.1590/0037-8682-0232-2024

**Published:** 2025-01-27

**Authors:** Thiago Elias Ferrari Khouri, Gabriel Chahade Sibanto Simões, Adriano Fregonesi

**Affiliations:** 1 Universidade Estadual de Campinas, Departamento de Urologia, Campinas, SP, Brasil.

Paracoccidioidomycosis (PCM) is a fungal disease endemic to Latin America. PCM rarely involves the male genital tract and generally occurs in the disseminated form of the disease, in which other manifestations coexist. It can affect the penis, epididymis, testicle, and prostate^1^ ^,^ ^2^ ^,^ ^3^.

**FIGURE 1:**
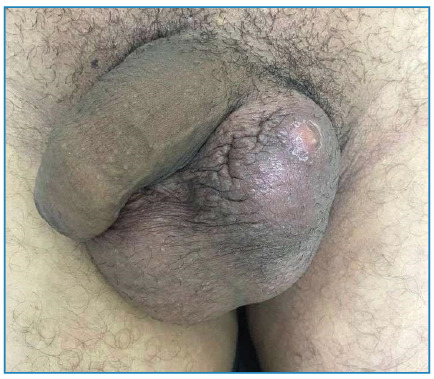
Physical examination.

A 53 year-old-man from a rural area presented with rapid and progressive growth of the left testicle, associated with local pain (Figure 1). In 2021, he was diagnosed with PCM and used itraconazole irregularly.

Initially,ultrasonography confirmed the diagnosis, without improvement after antibiotic therapy. The tumor markers were negative. Therefore, orchiectomy was performed.

Intraoperatively, the testis was soft and reduced in size. After opening the albuginea, a stroma with a necrotic appearance and purulent secretion and an enlarged and hardened epididymis were observed, with the presence of a large amount purulent secretion.

Anatomopathological examination revealed signs of PCM in the testes and epididymis (Figure 2A, B).

**FIGURE 2A:**
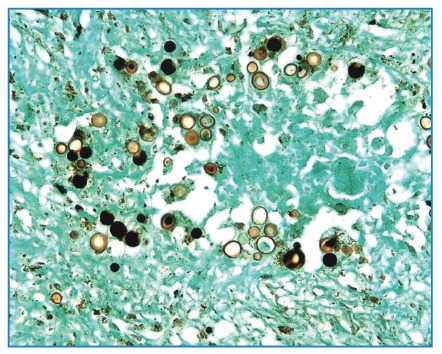
*P. brasiliensis* esporulation (40x, Grocott-Gomori).

**FIGURE 2B:**
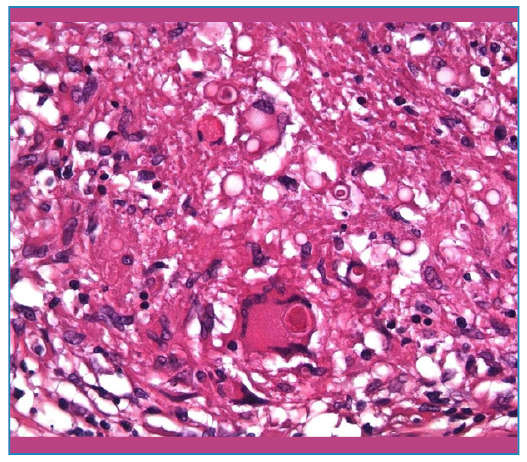
Epithelioid macrophages, Langhans giant cells, and spores of *P. brasiliensis* within a granuloma (40x, Hematoxylin-eosin).

PCM of the male genital tract is rare, and according to recent studies, it appears to be the least affected site^3^.

The presentation is similar to that of a painful lesion, and the differential diagnosis includes squamous cell carcinoma, testicular tumors, leishmaniasis, syphilis, tuberculosis, orchiepidymitis. Diagnosis is made via biopsy, and direct microscopy with potassium hydroxide preparations rapidly identified the fungus. The first-line treatment was itraconazole at a dose of 200 mg/day for 9-18 months^4^.
